# EEG Single-Trial Detection of Gait Speed Changes during Treadmill Walk

**DOI:** 10.1371/journal.pone.0125479

**Published:** 2015-05-01

**Authors:** Giuseppe Lisi, Jun Morimoto

**Affiliations:** Dept. of Brain Robot Interface, ATR Computational Neuroscience Laboratories, Kyoto, Japan; Duke University, UNITED STATES

## Abstract

In this study, we analyse the electroencephalography (EEG) signal associated with gait speed changes (i.e. acceleration or deceleration). For data acquisition, healthy subjects were asked to perform volitional speed changes between 0, 1, and 2 Km/h, during treadmill walk. Simultaneously, the treadmill controller modified the speed of the belt according to the subject’s linear speed. A classifier is trained to distinguish between the EEG signal associated with constant speed gait and with gait speed changes, respectively. Results indicate that the classification performance is fair to good for the majority of the subjects, with accuracies always above chance level, in both batch and pseudo-online approaches. Feature visualisation and equivalent dipole localisation suggest that the information used by the classifier is associated with increased activity in parietal areas, where mu and beta rhythms are suppressed during gait speed changes. Specifically, the parietal cortex may be involved in motor planning and visuomotor transformations throughout the online gait adaptation, which is in agreement with previous research. The findings of this study may help to shed light on the cortical involvement in human gait control, and represent a step towards a BMI for applications in post-stroke gait rehabilitation.

## Introduction

Previous EEG studies have found an increased cerebral activity during walking or its motor preparation. The sensorimotor area is significantly activated during isolated movements, such as leg or foot movements, that represent a part of human locomotion [1, 2]. Moreover, the activity in the prefrontal cortex is enhanced when preparing for and performing obstacle stepping on a treadmill compared to normal walking [3]. Other studies have suggested that the EEG signal contains information associated with the gait phase [4], and with the kinematics of the legs [5]. Increased cerebral activity (i.e. suppressed alpha and beta activity) was found over foot sensorimotor areas during active walking compared to passive walking; and suppressed alpha and beta activity over premotor and sensorimotor areas was found during walking as opposed to a rest condition [6]. Additionally, significant power perturbations in the EEG lower gamma band (25–40 Hz) were observed over the premotor cortex and over the foot area of the primary motor cortex, during active and robotic assisted treadmill walk [6–8]. Moreover, it has been shown that midline cortical areas display increased EEG theta band activity during walking on a balance beam compared to treadmill walk [9]. According to a recent study [7], premotor and parietal areas display increased activity during treadmill walk with adaptive virtual environment, as compared to walking in front of a mirror or with movement unrelated feedback. Other studies, based on fNIRS, have found encouraging evidence of cortical involvement during walking related tasks. In the process of accelerating from a standing-still state (i.e. gait initiation) the prefrontal and premotor cortices activity increases, as compared to a standing-still rest condition; while steady state walking does not elicit cortical activation [10]. Furthermore, the prefrontal cortex is more active during precision stepping as compared to normal gait [11].

Among the most critical issues when recording the EEG signal during highly dynamical tasks, such as locomotion, there are artifacts [12], of both physiological or non-physiological origin. With regards to the former typology, electrooculography (EOG) and electromyography (EMG) activities are considered among the most critical sources of interference in brain computer interface (BCI) systems [13]. Temporal muscle activations typically induce 20–60 Hz activity at temporal electrodes, which is maximal at 30 Hz; while eye movements produce strong low frequency (1–4 Hz) activity at frontal electrodes [13, 14]. It has been shown that such artifacts can be removed by the application of independent component analysis and by thresholding of higher order statistics [14, 15] and spectral perturbation magnitude [16]. Non-physiological artifacts, that originate from outside the human body (e.g. power-line noise), can be typically avoided by properly filtering the signal. However, muscle and mechanical artifacts are enhanced during locomotion and running, due to head movements and shocks undergone by the whole body at each step. Castermans et al. [17] found that the EEG signal may be polluted up to 15 harmonics of the fundamental stepping frequency and in high-gamma frequency bands extending up to 150 Hz. Bertrand et al. [18], observed that during different types of motions, including walking and jumping, most of the artifact energy is present in the low frequencies (< 5Hz) of the EEG signal.

Previous studies have proposed different methods to reduce the effect of artifacts on the signal recorded during treadmill walking. Severens et al. [19] cleaned EMG artifacts by means of Canonical Correlation Analysis. Bertrand et al. [18] proposed a multi-channel linear prediction filter, based on contact impedance measurements, to remove motion artifacts. Petersen et al. [20] applied Independent Component Analysis (ICA) after visual inspection of the EEG signal. In 2010, Gwin et al. [12] employed ICA and a template subtraction method, in order to remove movement artifacts from the EEG signal of a P300 task, executed during locomotion at different speeds and running. However, this subtraction approach would remove the information of interest if applied to the neural process associated with locomotion itself. Therefore, in their next study, Gwin et al. [4] proposed to directly remove artifactual independent components, based on the inspection of their power spectra and locations of their equivalent dipoles, computed by an inverse modelling method. For robotic assisted treadmill walk, Wagner et al. [6, 7] followed a similar rejection approach, however the subsequent EEG analysis was performed on a narrower band (3–40 Hz) as compared to Gwin et al. [4] (1–150 Hz). Given the significant amount of literature that employs ICA, in the current study we use an ICA-based automatic method, inspired by the works of Wagner et al. [6, 7], with slight modifications to automatically cope with known artifactual independent compoenents (i.e. ADJUST [21]) and with strong muscle activations [16].

According to the presented literature, different areas of the cortex are involved in the preparation and adaptation of gait, therefore we hypothesise that gait speed changes would increase the activity in some of these brain areas. Acceleration from a standing-still state (i.e. 0 Km/h) to 3, 5 and 9 Km/h has already been investigated in an fNIRS study [10]. In [10], the brain activity baseline, used to analyse the three types of acceleration, was always the resting state (i.e. 0 Km/h). However, Wagner et al. [6] showed that, during walking, the EEG signal displays a significant power decrease compared to a rest condition. Therefore, the question is whether acceleration and deceleration can be detected, even when starting from non standing-still conditions (i.e. 1 and 2 Km/h). To the authors knowledge, this is the first time that the EEG signal is measured during volitional gait acceleration from, or deceleration to, a standing-still steady state (i.e. 0 Km/h) and a non standing-still steady state (i.e. 1 and 2 Km/h). Increased brain activity during gait speed changes may be exploited by a BMI system that classifies between the two classes *constant-speed* and *change-speed*.

## Methods

We designed a treadmill walk experiment in which EEG signals were collected while healthy subjects accelerated and decelerated their gait within the same session. In order to allow subjects to perform volitional speed changes, the treadmill controller dynamically modified the speed of the belt according to the subject’s linear speed, monitored by a position sensor. In this way, within the same experiment the subjects could dynamically change their speed between 0, 1 and 2 Km/h. After describing the experimental design in section 3, we introduce the steps necessary to train and evaluate an EEG classifier that is able to discern between the *constant-speed* and the *change-speed* conditions. Specifically, in section 4 we describe a batch classification, since the associated statistical analysis (section 4.9), feature visualization (section 5) and artifact analysis (section 6) are straightforward. Subsequently, we extend the study to a pseudo-online analysis (section 7), which aims to analyze the evolution of the decoder’s output in time and, thus, to estimate the performance for future online applications.

### 1 Participants and ethics statement

Eight healthy volunteers, aged 25±2.5 and without neurological disorders or locomotor deficits, took part in the experiment. Subjects gave written informed consent for the experimental procedures, which were approved by the ATR Human Subject Review Committee.

### 2 Treadmill control and sensors setup

The treadmill (Woodway USA, Inc) used in this study is endowed with a serial interface for external control commands. We designed a real-time system which monitored the subject’s speed on the treadmill by means of a linear encoder (Microtech Laboratory Inc., MLS-30 series), and sent commands to the treadmill if the subject’s speed exceeded a threshold (±0.12 m/s). Specifically, if the speed of the subject overcame the negative or the positive threshold, the belt respectively increased or decreased speed of 1 Km/h.

**Fig 1:**
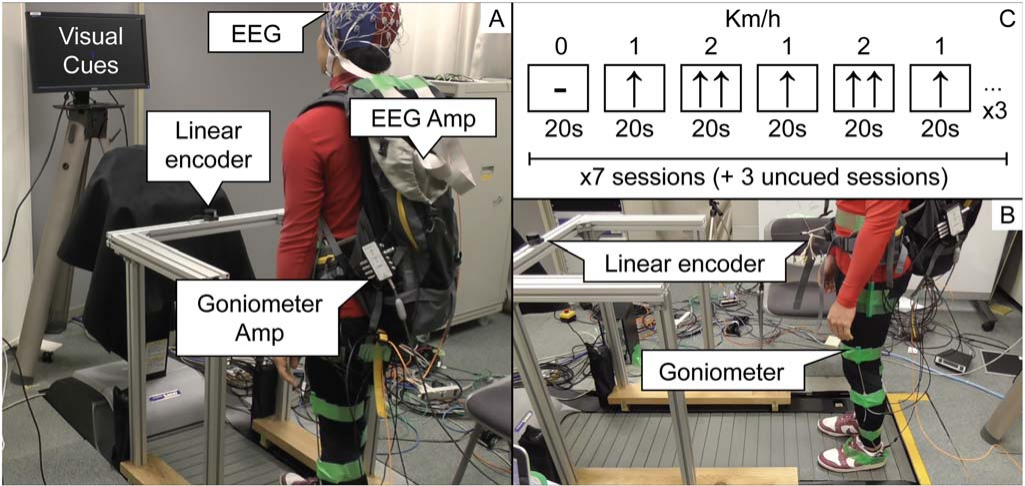
Experimental setup. In panel A (left), it is represented the back view of the experiment. The screen that presents visual cues is positioned in front of the subject at the height of the eyes. The EEG and goniometer amplifiers are held by means of a backpack, carried by the subject, and the EEG cables are fixed by tape to the top of the backpack to avoid oscillations. The linear encoder is placed at belt height by means of an aluminium frame. In panel B (right-bottom), we show the side view of the experiment. It is possible to see that one extremity of the linear encoder is attached to the aluminium frame and the other one is tied to the backpack belt. The goniometer sensors are attached to the hip, knee and ankle of both legs. In panel C, it is represented one of the three repetitions of visual cues that are shown to the subject during one session.

The EEG signal was recorded at a sampling rate of 2048 Hz, by means of a 64-electrodes Biosemi Active Two system, zero-referenced using the Common Mode Sense (CMS) and the Driven Right Leg (DRL) electrodes. Moreover, the angles of the hip, knee, and ankle joints were recorded by means of a goniometer sensors (Biometrics Ltd). All the sessions were recorded on video in order to verify possible anomalies in the collected data (Panels A and B in Fig 1).

### 3 Experimental design

The experiment was composed of 10 sessions of 380 seconds each. During 7 out of 10 sessions, visual cues instructed the subjects about when to modify their speed. The visual cues (Panel C in Fig 1) were designed to generate, within the same session, 19 blocks (20 s each) ordered by speed (Km/h) as follows: 0-1-2-1-2-1 (×3, followed by rest). The visual cues changed at the beginning of each block and were kept throughout its duration. Subjects were instructed to modify their speed based on the visual cues, but without any strict time constraint, since their movements were monitored by the linear encoder.

In the remaining 3 out of 10 sessions, no visual cues (*uncued*) were presented to the subjects, who autonomously decided the timing of the gait speed modification, while following the order previously described. Also the *uncued* sessions were terminated after 380 seconds, even if the block repetitions were not completed, in order to avoid a long and uncomfortable duration of the session. Moreover, an *uncued* session was performed after two or three *cued* sessions, so that EEG non-stationarities [22] would not affect one condition more than the other.

The *cued* condition is necessary in order to control the amount and type of speed changes performed within 380 s of a session, and to help subjects to get acquainted with the experiment. *Uncued* sessions are used to validate that the decoded brain activity is actually related to the gait speed changes and not to the visual cues.

Before starting the experiment, subjects were asked to get used to the treadmill control system for a few minutes. In order to facilitate the learning phase we created a positioning scheme that roughly corresponded to the three levels of speed: at 0 Km/h stand at the rear of the treadmill, at 1 Km/h walk at the centre of the treadmill and at 2 Km/h walk at the front of the treadmill. Moreover, subjects were instructed to always look straight, step on the treadmill as gently as possible, avoid abnormal arm and neck movements, blink normally and, in the *uncued* sessions, avoid counting time.

The adaptation of the treadmill to the new speed had to be performed gradually, i.e. 4 s during acceleration and 8 s during deceleration, in order to avoid jerks that could have put the subjects in danger of falling, or could have led to strong muscle artifacts associated with sudden balancing motions. The data collected by goniometers confirm that, on average (±*SD*, standard deviation), the time to reach steady speed was 4.62*s*±1.09 during acceleration and 8.19*s*±1.86 during deceleration. Moreover, we quantify the duration of the walk after reaching steady speed, separately for the *cued* and *uncued* sessions. In the former case, the steady state walk lasted 15.5*s*±1.2 after acceleration, and 11.5*s*±1.97 after deceleration; while in the *uncued* sessions, the duration of the steady state walk was 17.4*s*±5.35 after acceleration, and 12.8*s*±6.51 after deceleration. The larger SD measured in the *uncued* sessions is in agreement with the expectations, since subjects decided autonomously the duration of the steady speed phases.

### 4 EEG classifier

**Fig 2:**
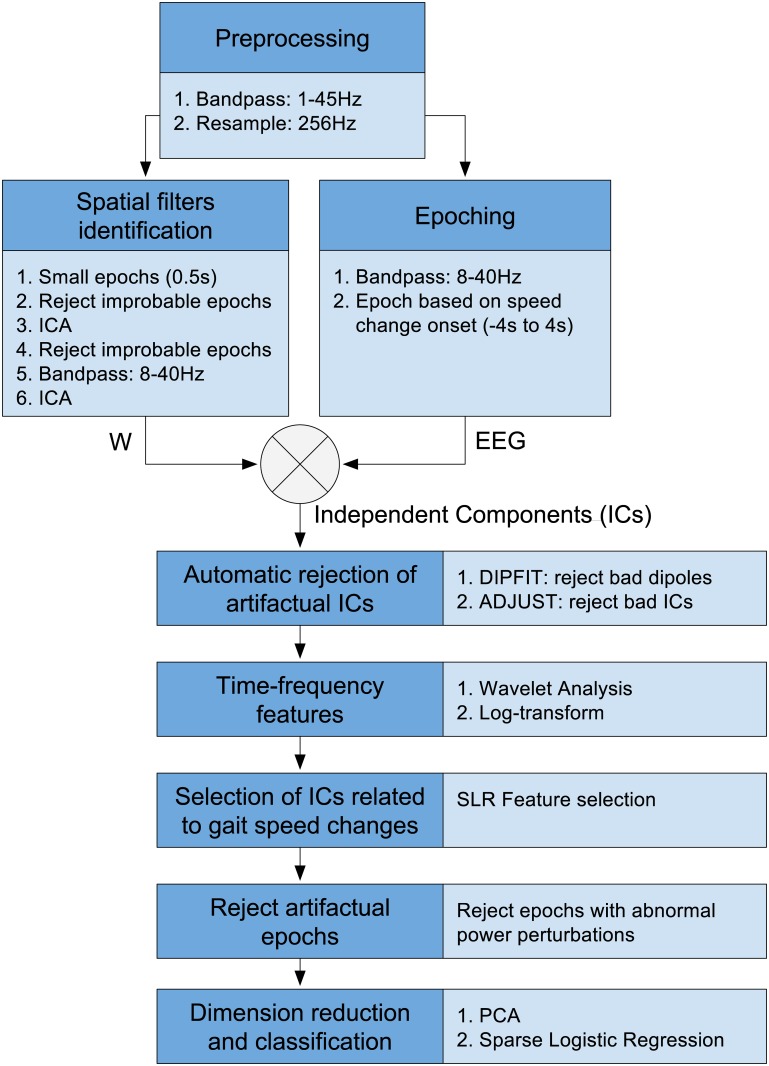
Classification algorithm. The light blue boxes contain the details of each step.

Individually for each subject, a batch classifier is trained to classify the EEG signal associated with constant speed walking or steady state (*constant-speed* class), against the one related to gait acceleration or deceleration (*change-speed* class). The methodology for training and testing the classifier was presented in our recent study [23]. In the current work we make use of the decoder based on Infomax ICA, since this technique has been extensively tested, in previous literature, both for artifact rejection [14] and unsupervised feature extraction [24]. In this section we summarize the training algorithm (Fig 2), with slight modifications in order to explicitly cope with gait-related motion artifacts. In order to assess the classifier performance, a 10-fold cross validation procedure is carried out; where, in each repetition, 9 out of 10 sessions are used for training, and the remaining one for testing.

#### 4.1 Preprocessing

All the 64 channels of the training and test sets are bandpass filtered in the range from 1 Hz to 45 Hz, by means of the EEGLAB function *pop_eegfiltnew*[25, 26]. This function designs a Hamming-windowed zerophase FIR filter with optimized order [27]; which, in the specific case, is automatically set to 6761, with cutoff frequencies 0.5 Hz and 45.5 Hz attenuated at -6 dB. Subsequently, the signal is resampled at 256 Hz, and used as input both for spatial filter identification (Section 4.2) and epoch extraction (Section 4.3).

#### 4.2 Spatial filter identification (ICA)

In order to decompose different neural and artifactual sources [28, 29], Infomax ICA is computed on the preprocessed EEG signal of the training set. Spatial filters of improved quality are achieved utilizing the algorithm proposed by the EEGLAB authors [14], in which ICA is computed two times, interspersed with two steps of outliers rejection. In detail, for automatic artifact rejection, the EEG signal is partitioned into small segments of 0.5 s, and a segment is removed if its probability distribution exceeds the average distribution by 5±*SD*. The next step is to compute ICA a first time; segments are further rejected based on the probability distribution of the independent component (IC) projections; the ‘clean’ EEG segments are bandpass filtered between 8 and 40 Hz (FIR filter order 425; cutoff frequencies 7 Hz and 41 Hz at -6 dB); and finally, ICA is computed a second time. Bandpass filtering, before the final ICA, is added to the original algorithm because it can improve the performance of ICA algorithms [24, 30]. Once the ICA unmixing matrix is computed, it is applied to the EEG signal as explained in the next section.

#### 4.3 Epoch extraction

**Fig 3:**
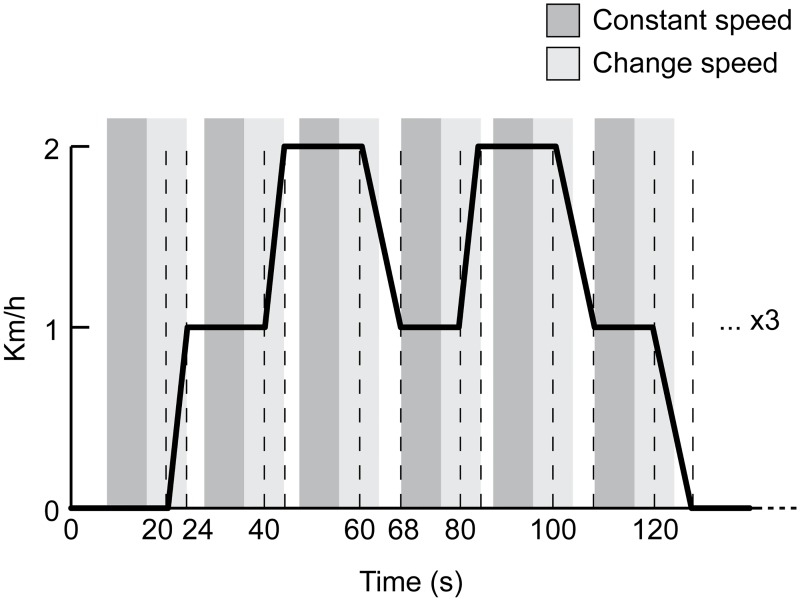
Epochs with respect to gait speed profile. The ideal profile of the gait speed, associated with one of the three repetitions within one session, is visualised. In order to reduce the effect of jerks on the experiment, it is important to perform gradual speed changes of the treadmill, therefore the time required for acceleration is 4 s and for deceleration it is 8 s. Epochs corresponding to the *change-speed* class are represented by a light grey area that is centred with respect to the *change-speed* onset (0 s) and ranges between -4 s and 4 s. While the epochs related to the *constant-speed* class range between -12 s and -4 s and are coloured with a darker grey. White spaces, located before the constant speed epochs, represent period of constant speed that are not taken into consideration for classification.

The preprocessed continuous EEG signal (Section 4.1) is further bandpass filtered between 8 and 40 Hz (filter parameters as in Section 4.2). Epochs are extracted with respect to the gait speed change onset (Fig 3): if we refer to the instant at which acceleration or deceleration is detected as the time 0 s, the epochs associated with the *change-speed* class range between -4 s and 4 s, while the ones related to the *constant-speed* class range between -12 s and -4 s. The epochs length is fixed in order to match the constant number of features in input to the classifier, and is selected after careful preliminary analysis regarding the goniometer data, presented in the last paragraph of section 3. Specifically, the window is designed to cover as much data as possible, while avoiding overlapping between *constant-speed* epochs and the final part of the previous speed change (see Fig 3). Large epochs are suitable to discover any possible difference between the two classes, before and after the change of speed, throughout the period of speed adaptation. In this way, we also keep the dataset balanced, which makes the classification performance assessment straightforward. The epoch extraction step produces 18 trials per class, for each *cued* session; and on average 15.9±2 trials per class for each *uncued* session. Within a cross-validation fold, 1 out of 10 sessions is kept as test set and the remaining ones are used as training set. Finally, the epochs of both the training and test sets are multiplied by the ICA unmixing matrix, obtained at the previous step (Section 4.2).

#### 4.4 Automatic rejection of artifactual spatial filters

The automatic rejection of artifactual spatial filter is performed on the training dataset, while the test set is adjusted accordingly. In particular, a singe dipole source localisation algorithm with a BESA 4-shell spherical model [31], implemented in EEGLAB [32], is run on the independent component scalp maps, in order to extract equivalent current dipoles. The residual variance (r.v.) of dipole fitting is used to reject independent components of poor quality (*r*.*v*. > 10% [6]). Furthermore, an independent component is automatically rejected if its dipole is located outside of the head, close to the skull or in the cerebellum, utilizing the following heuristics: distance from the center of the spherical model larger than 70% of the sphere radius, or z-coordinate smaller than 0 mm. These thresholds are chosen in such a way that artifactual components with inaccurate dipole localization would still [33, 34] be correctly removed. Finally, in order to explicitly remove components associated with eye movements, we use the ADJUST plugin for EEGLAB [21], an automatic method that combines stereotyped artifact-specific spatial and temporal features, optimized to capture blinks, eye movements, and generic discontinuities.

#### 4.5 Time-frequency analysis: the wavelet transform

The Morlet Wavelet transform [35] is employed to extract time-frequency features from the retained independent components of both the training and test set. For each epoch, a wavelet coefficient matrix with 50 time points (i.e. -3789 to 3789 ms) and 20 log-spaced frequency bins (i.e. 8–40 Hz) is computed for the *i*-th independent component. Each of the 50 time windows are composed of 107 samples (418 ms) overlapped by 68 samples (266.5 ms); while the number of cycles ranges from 3 to 7.5, with an increment of 0.5 from the lowest to the highest frequency. The resulting coefficients are squared to get the spectral power and the 10log_10_ transformation is computed to obtain the final time-frequency representation.

#### 4.6 Selection of ‘neural’ spatial filters associated with gait

Some of the spatial filters retained after artifact rejection, might still not be related to gait (e.g. auditory components). In order to avoid overfitting and to obtain physiologically plausible results, it is necessary to reduce the number of spatial filters to the ones that are strictly indispensable for the classification problem. To accomplish this goal, we apply a method, proposed in our previous work [23], that rejects independent components based on the number of features selected by sparse logistic regression (SLR) [36]. In the current work, the rejection threshold is defined by using the Scree Test Acceleration Factor [37], which is more effective than simple thresholding based on standard deviation.

#### 4.7 Rejection of artifactual epoch

The spectrograms associated with the retained spatial filters are analysed for abnormal muscle artifact rejection. In particular, an epoch is rejected if the power perturbation, in the 20–40 Hz band, deviates by +25 or -100 dB from the baseline [16] at least for one independent component. This process removed on average 0.07% of the trials along cross-validation folds and subjects. Such a low rejection rate is due to the fact that, at this point, only the features derived from ‘non-artifactual’ ICs are taken into consideration. After the rejection of artifactual epoch, the feature vector of the *i*-th trial is obtained by the concatenation of ‘clean’ time-frequency coefficients, computed from ‘non-artifactual’ independent components.

#### 4.8 Dimensionality reduction and classification

Principal Component Analysis (PCA) is applied to the training set, in order to reduce redundancy and dimensionality of the time-frequency features (i.e. 90% of percentage of variance [38]). The resulting projection matrix is also used to derive the principal components of the test set, which was previously normalized by subtracting the mean of the training set.

The reduced feature vectors of the training and test sets are used, with the respective labels (i.e. *constant-speed* vs. *change-speed*), to train and evaluate the definitive sparse logistic regression classifier [36]. The confusion matrix, obtained by validating the classifier with the test set, is combined with the confusion matrices of the other cross-validation folds, as explained in section 4.9.

#### 4.9 Performance assessment

Individually for each subject, the confusion matrices of all the cross-validation folds’ test sets are summed up. The resulting confusion matrix is class-wise normalized, and the mean and SD of the main diagonal are computed, to obtain an intuitive estimate of the 10-fold cross-validated classification performance (Table 1).

**Table 1:** Classification accuracy.

	S 1	S 2	S 3	S 4	S 5	S 6	S 7	S 8
Accuracy (%)	71±1.8	72±2.5	58±3.8	66±0.89	77±0.97	77±5.4	81±6.6	79±5

Individual 10-fold cross-validated classification accuracy between the *constant-speed* class and the *change-speed* class.

For further comparisons, the classification performance is assessed by computing the Cohen’s Kappa [39], which is a measure commonly employed in BCI single-trial classification studies [40, 41]:

$$\begin{array}{c} k=\frac{p-p_{0}}{1-p_{0}} \end{array}$$ (1)

In the equation, *p* is the proportion of observed agreements, and *p*_0_ is the proportion of agreements expected by chance. Perfect agreement between the true target labels and the predicted ones is represented by *k* = 1, while agreement no better than that expected by chance is indicated by *k* = 0. The advantages of using Kappa are that it considers the distribution of wrong classifications (i.e. the off-diagonal elements of the confusion matrix) [42], and it has already been used for within-subject statistical comparisons of classification performances, in previous BCI studies [43, 44]. In our analysis, *k* is computed on the summation of confusion matrices across cross-validation folds, so to obtain a cross-validated measure. Moreover, *k* is significant (*α* = 0.05) if its adjusted Wald confidence interval lower bound is *k*_*l*_ > 0 [41]. We confirmed with the authors of [41] that the formulas of the original paper (17.18 and 17.19) contain a misprint: the square root should be over the term (N+4). Therefore the corrected version of *k*_*l*_ is rewritten here:

$$\begin{array}{c} k_{l}=\hat{k}-z_{1-\alpha /2}\sqrt{\frac{\hat{p}\left(1-\hat{p}\right)}{\left(N+4\right){\left(1-p_{0}\right)}^{2}}} \end{array}$$ (2)

where

$$\begin{array}{c} \hat{k}=\frac{\hat{p}-p_{0}}{1-p_{0}} \end{array}$$ (3)

$$\begin{array}{c} \hat{p}=\frac{C+2}{N+4} \end{array}$$ (4)

and where *z*_1−*α*/2_ is the quantile of the 95% confidence interval of the standard normal distribution, *N* is the number of trials and *C* is the number of correctly classified trials. In the results, a non significant Kappa is reported with an asterisk.

In order to rule out the possibility that the classifier uses features associated with the visual stimuli, we combine the confusion matrices of the *cued* test sets, separately from the *uncued* test sets, and we compute the respective Kappa scores. A within-subject comparison between the *K*_*uncued*_ and *K*_*cued*_ is performed by paired sample t-test (Table 2).

**Table 2:** Cued vs. Uncued conditions.

Condition	S 1	S 2	S 3	S 4	S 5	S 6	S 7	S 8	p-value
Cued	0.47	0.47	0.18	0.31	0.52	0.52	0.57	0.56	0.71
Uncued	0.35	0.37	0.062*	0.34	0.59	0.59	0.78	0.65	

Within-subject comparison of the Kappa scores associated with the *cued* and *uncued* conditions. Non significant Kappa scores are marked with *. The p-value of the paired sample t-test is also reported.

Furthermore, we verify that every subclass of *change-speed* (i.e. 0 → 1, 1 → 2, 2 → 1, 1 → 0) was classified as *change-speed* above chance level. To do so, for each subclass we compute the cross-validated Kappa score associated with its labels and with the labels of the preceding constant speed (e.g. 1 → 2 vs. 1). In order to check whether any subclass has a significantly better performance across subjects, we compute a repeated measurements within-subject one-way ANOVA (*α* = 0.05), with factor *subclass* (Table 3). If the sphericity assumption was not met, the resulting F-values were adjusted by the Greenhouse-Geisser method. *Post hoc* testing was performed by Bonferroni corrected paired t-tests.

**Table 3:** Subclasses performace.

Subjects	0 → 1	1 → 2	2 → 1	1 → 0
S1	0.7	0.33	0.33	0.54
S2	0.53	0.37	0.56	0.29
S3	0.27	0.17*	0.16*	0*
S4	0.56	0.15*	0.45	0.15*
S5	0.56	0.51	0.54	0.56
S6	0.36	0.55	0.57	0.62
S7	0.82	0.53	0.6	0.69
S8	0.53	0.5	0.73	0.52
Avg	0.54	0.39	0.49	0.42

Within-subject comparison of the Kappa scores associated with the subclasses of the *change-speed* class (e.g. 1 → 2 represents the class where the speed changes from 1 to 2 Km/h). Non significant Kappa scores are marked with *.

Moreover, we investigate whether the brain activity preceding the speed change onset could be sufficient in order to classify. To do so, we retrain the binary classifier (10-fold cross-validated), using only the time-frequency features computed with the EEG signal preceding 0 s (i.e. from -3789 to -211 ms), for each trial. This analysis is addressed as *pre-onset*, as opposed to the original procedure (i.e. -3789 to 3789 ms) that is named *full-epoch*. A within-subject comparison between the *full-epoch* and the *pre-onset* Kappa is performed by a paired sample t-test, both for the *cued* (Table 4) and *uncued* (Table 5) conditions.

**Table 4:** Pre-onset analysis for the cued condition.

Classifier	S 1	S 2	S 3	S 4	S 5	S 6	S 7	S 8	p-value
Pre-onset	0*	0.18	0.062*	0.046*	0.3	0.15	0*	0.14	0.0003
Full-epoch	0.47	0.47	0.18	0.31	0.52	0.52	0.57	0.56	

Within-subject comparison, of the *pre-onset* and *full-epoch* Kappa score, for the *cued* condition. Non significant Kappa scores are marked with *. The p-value of the paired sample t-test is also reported.

**Table 5:** Pre-onset analysis for the uncued condition.

Classifier	S 1	S 2	S 3	S 4	S 5	S 6	S 7	S 8	p-value
Pre-onset	0.082*	0.033*	0*	0*	0.31	0*	0.13*	0.17*	0.0008
Full-epoch	0.35	0.37	0.062*	0.34	0.59	0.59	0.78	0.65	

Within-subject comparison of the *pre-onset* and *full-epoch* Kappa score, for the *uncued* condition. Non significant Kappa scores are marked with *. The p-value of the paired sample t-test is also reported.

### 5 Feature visualisation

**Fig 4:**
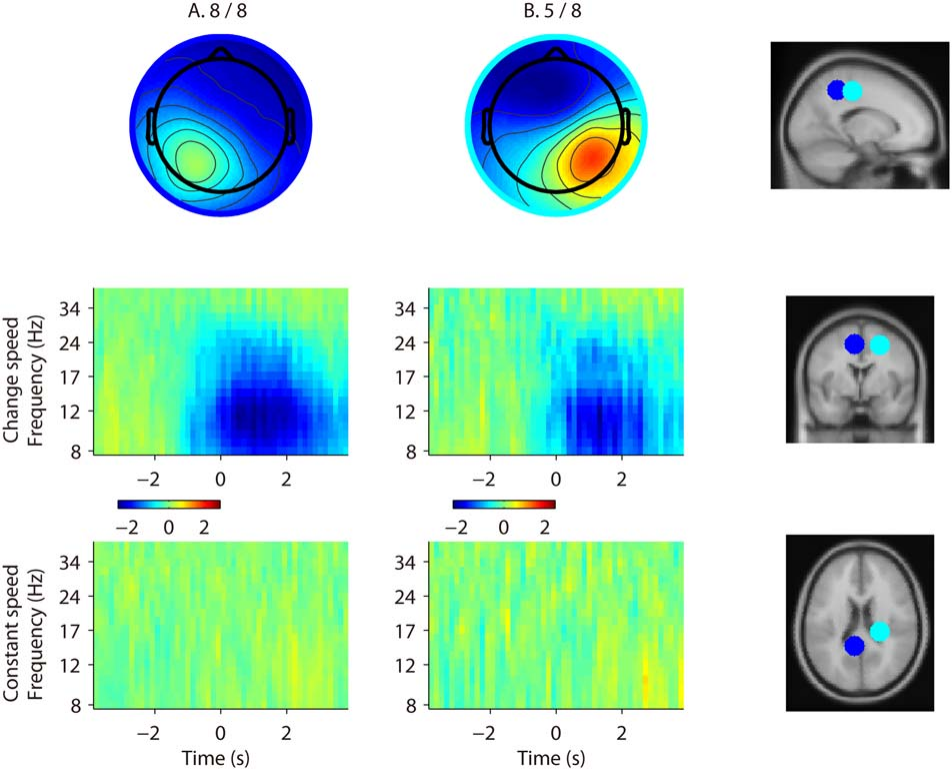
Feature visualization. The scalp maps of each cluster are visualised in the top row of the figure. Below each cluster’s scalp map, we show the ERSP corresponding to the *change-speed* and *constant-speed* classes. For each cluster, ERSPs are normalised using the mean log spectrum over the whole epoch length of the *constant-speed* class, and the respective color axis is visualised. On the right side, the dipoles corresponding to cluster centroids are displayed on the standard MNI brain model in sagittal, coronal and axial view. The colour of each dipole corresponds to the colour of the circle surrounding the respective cluster scalp map. We observe that both clusters are characterized by ERD accompanying the change of speed.

In order to obtain a compact representation of the features used by the batch classifier, we cluster the retained independent components (IC) across all the subjects and cross-validation folds, by a robust k-means algorithm (*k* = 4; and outlier rejection *SD* = 5), implemented in EEGLAB [25]. For this purpose, each IC is represented by a vector that contains the 3D coordinates of its dipole and the principal components (90% of the variance) of its scalp map. If a subject contributes to an IC cluster with less than 40% of his total cross-validation folds, he is removed from the IC cluster, and only IC clusters composed of two or more subjects are presented. The scalp map representing a cluster is obtained by averaging the scalp maps of its components (Fig 4).

Moreover, for every IC cluster, we visualise (Fig 4) the equivalent current dipole and the Event Related Spectral Perturbation (ERSP [45, 46]) associated with the *change-speed* and *constant-speed* classes. The ERSPs of both classes are baselined by subtracting the mean log spectrum over the whole epoch length of the *constant-speed* class, in order to highlight possible differences. The dipole centroid of a cluster is computed by averaging the 3D coordinates of its components’ equivalent dipoles [32]. Only for visualization and approximate localization, each centroid is automatically shifted towards the dorsal direction until it intercepts the ‘cortex’ sphere (i.e. 58% of the BESA sphere). This is done to correct for dipole modelling inaccuracies [33, 34, 47–49]. The Talairach coordinates of each cluster centroid and the respective nearest region of grey matter in the brain [50, 51] are also reported in Table 6, for interpretation and comparison with related studies.

**Table 6:** Cluster location.

Cluster	Talairach coordinates (x,y,z)	Location (Brodmann Area)
A	-6, -42, 48	Parietal cortex (BA7)
B	24, -25, 46	Primary motor cortex (BA4)

For each cluster, the approximate Talairach coordinates of the centroid and the corresponding nearest region of grey matter in the brain are shown.

### 6 Artifact analysis

#### 6.1 EEG correlation

**Fig 5:**
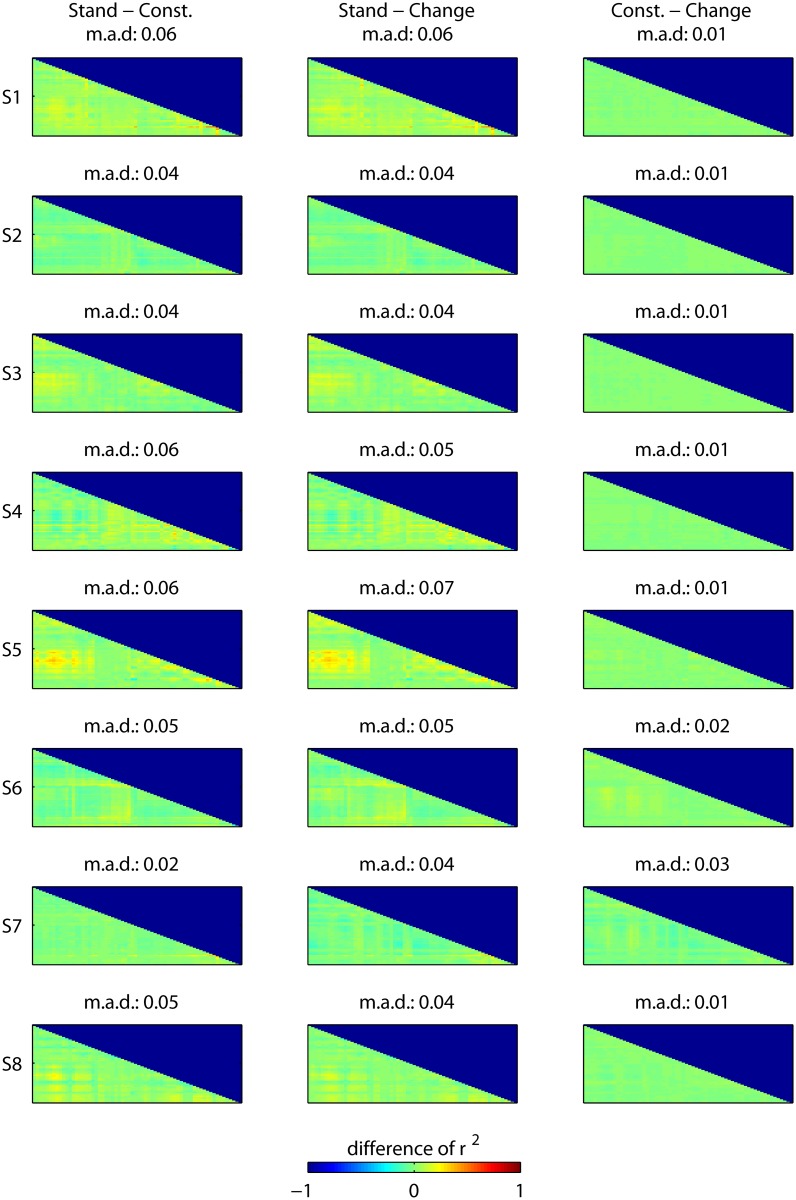
EEG correlation analysis. This figure shows, for each subject, the difference between every experimental condition, in terms of correlation (*r* ^2^) between the EEG channels. This is done in order to prove that motion artifacts are not affecting one condition more than the other. In this case, the standing still condition (i.e. 0 Km/h) is separated from the *constant-speed* condition (i.e. 1 and 2 Km/h), since we expect the former to be free of motion artifacts and a suitable ‘gold standard’. The EEG is bandpass filtered between 1–45 Hz, the correlation matrices of every trial are squared (*r* ^2^) and averaged separately for each condition. Then, the difference of *r* ^2^ matrices is computed and plotted for every possible combination of conditions. In figure, the order of the channels is, based on the International 10–20 system, Fp1, AF7, AF3, F1, F3, F5, F7, FT7, FC5, FC3, FC1, C1, C3, C5, T7, TP7, CP5, CP3, CP1, P1, P3, P5, P7, P9, PO7, PO3, O1, Iz, Oz, POz, Pz, CPz, Fpz, Fp2, AF8, AF4, Afz, Fz, F2, F4, F6, F8, FT8, FC6, FC4, FC2, FCz, Cz, C2, C4, C6, T8, TP8, CP6, CP4, CP2, P2, P4, P6, P8, P10, PO8, PO4, O2. Moreover, for every combination we provide the mean absolute difference (m.a.d.) between the *r* ^2^ matrices. This analysis does not highlight an increased correlation either for the *constant-speed* or the *change-speed* conditions, which suggests that the information used by the classifier is not associated with motion artifacts.

Motion artifacts may be stronger or weaker during gait speed changes, as compared to the constant speed condition. Assuming that motion artifacts would have a coherent effect across several channels, if not all electrodes, we would expect a generally higher correlation for a condition affected by motion artifacts. In order to exclude the possibility that one of the conditions is prone to artifacts more than the other, we compute the correlation matrix of the 64 EEG channels, in the frequency band 1–45 Hz, separately for each subject and condition. For the purpose of this analysis, the standing still condition (i.e. 0 Km/h) is separated from the *constant-speed* condition (i.e. 1 and 2 Km/h), since we expect the former to be free of motion artifacts and a suitable ‘gold standard’. The correlation matrices of every trial are squared (*r* ^2^) and averaged separately for each of the three experimental conditions (i.e. *standing still*, *constant-speed* and *change-speed*). The difference between every possible combination of conditions, in terms of *r* ^2^ matrix, is shown in Fig 5, separately for each subject. Moreover, we provide the mean absolute difference (m.a.d.) between *r* ^2^ matrices for each combination, in order to prove that the average correlation across channels does not change between conditions.

#### 6.2 Comparison of retained and rejected ICs in the time-frequency domain

**Fig 6:**
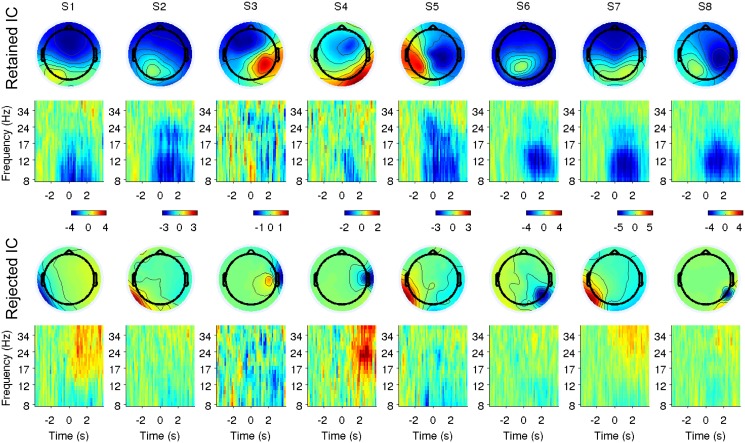
Comparison of retained and rejected ICs in the time-frequency domain. For each subject, the scalp maps and the ERSPs of the retained-rejected pair of independent components with the highest correlation in the time-frequency domain, and whose dipoles are localised in the posterior area, are visualised. In this case, each ERSP is normalised using the first 2.8 s (i.e. from -3789 to -1007 ms) as baseline. We do not observe any similarity between the ERSPs of the rejected and retained components.

For each subject, we verify that the classifier does not make use of gait-related artifactual information. This is carried out by comparing the ERSPs of retained components with those of rejected components outside the head or close to the skull. The rejected components that we consider for comparison are those related to dipoles with a distance from the centre of the spherical model larger than 90% of the radius, and with residual variance smaller than 10% (i.e. good quality scalp map [7]). Moreover, given the results of clustering (presented in Section 2), we focus on components whose dipoles are located in the posterior hemisphere (x-coordinate < 0). For each pair of rejected-retained components, the correlation coefficient among *change-speed* ERSPs (baseline from -3789 to -1007 ms) is computed. For each subject, the pair with the highest correlation coefficient is visualized along with the relative scalp map (Fig 6).

### 7 Extension to pseudo-online EEG classification

#### 7.1 Training and evaluation

In this section we assess the feasibility of applying the decoder with a shorter sliding window. This is useful in order to analyze the evolution of the decoder’s output in time and, thus, to estimate the performance for future online applications. For each subject, a 10-fold cross validation is carried out to train and assess the performance of the sliding window decoder.

The steps used to train the decoder are equivalent to the ones of the batch analysis (section 4), except for epoch extraction. The typical way to make classification more robust against time shifted signals is jittered training [52]. In the training set, 4 windows of length 2 s are used to extract epochs for a given trial. Specifically, the center of the sliding-window is shifted, with respect to the speed change onset (i.e. 0 s), from 0 to 1.5 s with a step of 0.5 s for the *change-speed* class, and from -10 to -7 s with a step of 1 s for the *constant-speed* class. The range and step of the *change-speed* class are selected based on the prior knowledge derived from batch feature visualization. On the other hand, the parameters for extracting the *constant-speed* epochs are chosen to span a wider range and, therefore, increase signal variability, while keeping the amount of epochs per class balanced.

In order to evaluate the decoder performance, the decision was to carry out a pseudo-online analysis, which allows for quantitative performance estimation. Therefore, for the test set, the centre of the sliding window is shifted, with a step of 0.5 s, from -1 to 3 s for the *change-speed* class, and from -9 to -5 s for the *constant-speed* class, where 0 s represents the speed change onset. Therefore, in the test set, we extract 9 epochs per trial. In this case, the ranges and steps are selected in order to cover as much signal as possible, while keeping the number of epochs per class balanced, for a fair comparison.

As a result, each epoch is 2 s long, therefore the wavelet coefficients (14 time and 20 frequency points) span from -0.79 to +0.79 s with respect to the center of the epoch. It should be noted that, in each fold, the independent components were not recomputed, but the ones from the corresponding fold of the batch analysis were reused. In the following sections, the term ‘trial’ indicates a group of 9 consecutive epochs sharing a label (i.e. *constant-speed* or *change-speed*).

#### 7.2 Performance assessment

**Fig 7:**
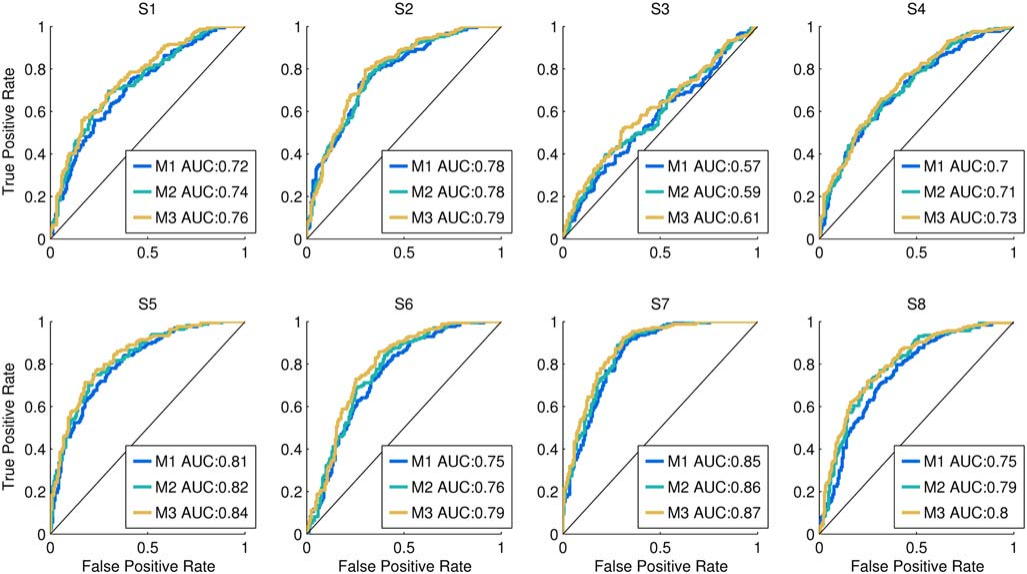
Pseudo-online classification performance. For each subject, the ROC computed with the all-or-nothing approach is displayed. The M2 and M3 curves are obtained by averaging, respectively, two and three consecutive outputs before computing the ROC, while for M1 no averaging is performed. In the legend, the AUC of each ROC is also provided. We observe that for every subject, the ROC curve lies above the chance level line (i.e. diagonal); and except for subject 3 the AUC has fair (0.7 ≤ *AUC* ≤ 0.79) to good (0.8 ≤ *AUC* ≤ 0.89) levels. Moreover, for every subject *AUC*_*M*1_ ≤ *AUC*_*M*2_ < *AUC*_*M*3_, even if the differences are small.

In the following paragraphs, a true positive is defined as a *change-speed* trial correctly classified, while a false positive is a *constant-speed* trial wrongly classified as *change-speed*. A logistic regression model, such as SLR, returns a probability that is subsequently transformed into a class by quantization (i.e. *constant-speed* class for *P* ≤ 0.5, *change-speed* class for *P* > 0.5). Depending on the threshold used to split the probability range into two classes, the number of true and false positive may vary. ‘Critical’ applications of the decoder may require low false positive rates (FPR), while other ones may be less strict. Therefore, to carry out a general evaluation, we assess performance based on the logistic model probability output, by means of the Receiver Operating Characteristic (ROC) and the respective Area Under the Curve (AUC). In detail, given that a single trial is represented by many shifted epochs, one false positive is sufficient to consider a whole *constant-speed* trial a failure. Similarly, one true positive is sufficient to determine that a whole *change-speed* trial is a success. Therefore, we adopt an all-or-nothing approach [53], where a whole trial is represented by the epoch with the highest output probability. After assigning a probability to every trial belonging to the test sets, the ROC and AUC are computed (Fig 7). In order to evaluate how the performance would change by combining consecutive outputs, we repeated this procedure after averaging the probabilities of 2 and 3 consecutive epochs, respectively, within each trial.

**Fig 8:**
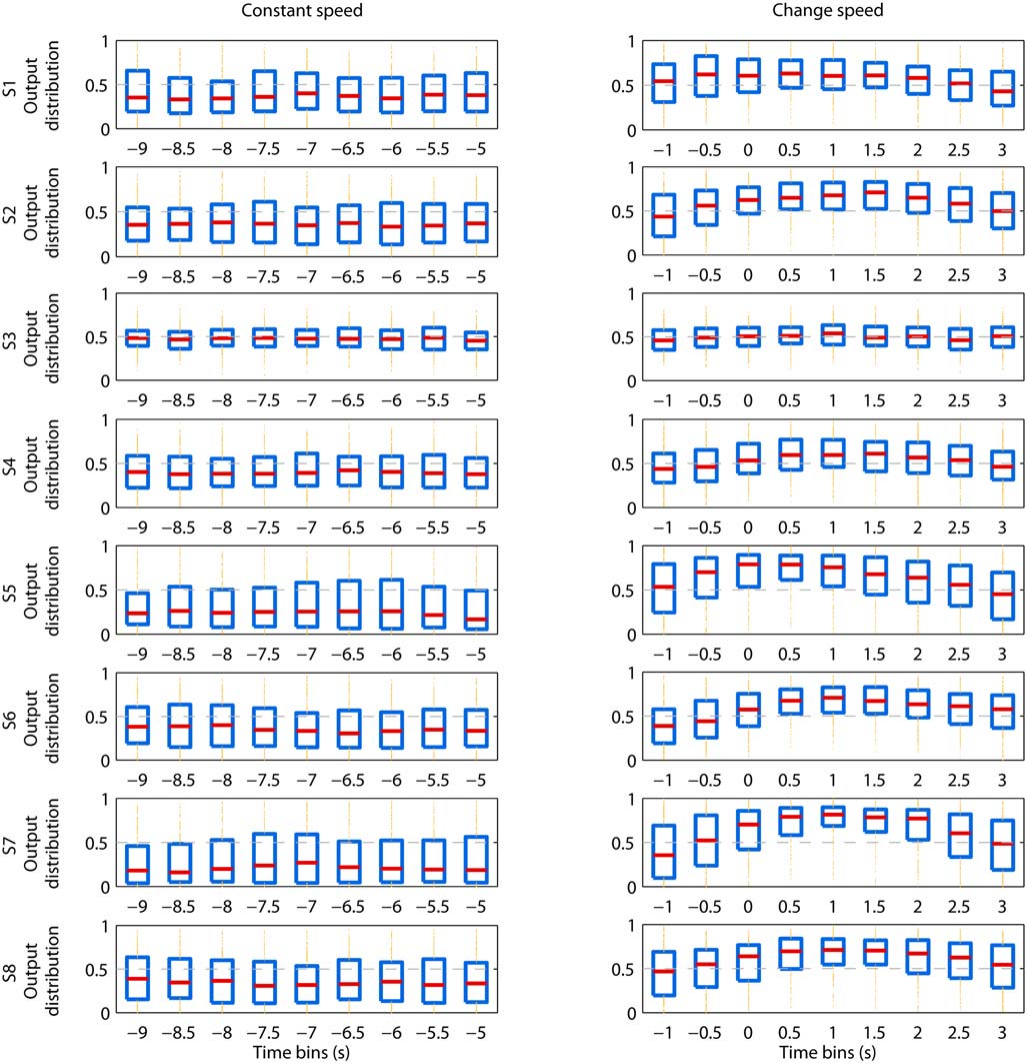
Temporal evolution of the decoder output. Each row represents one subject, while the left and right columns represent the *constant-speed* and *change-speed* conditions, respectively. Within each subplot the horizontal axis corresponds to the time bins spanned by the sliding window, with respect to the speed change onset 0 s; the vertical axis represents the decoder’s output distribution. The red horizontal lines are the median of the output, the edges of the blue boxes are the 25th and 75th percentiles, outliers are displayed with yellow dots above and below the percentile box, and threshold 0.5 is shown with a dashed gray line. For every subject, the median is always below the threshold 0.5 for the time bins belonging to the *constant-speed* class, while there is at least one time bin of the *change-speed* class that is above threshold. For the subjects with a fair to good AUC (Fig 7), the separation between the two classes can be observed not only for the median but also for the majority of the percentile box. Furthermore, in the *change-speed* condition, the time window that starts at -2 s and ends at 0 s (i.e. time bin -1 s) is characterised by a median close to threshold, for all subjects; which suggests that the decoder is able to detect the speed change, but not to predict it.

Moreover, we are interested in analyzing the temporal evolution of the decoder output. To do so, for each time bin, we compute the median of the decoder output, the 25th and the 75th percentiles and plot them in a boxplot, separately for the *constant-speed* and *change-speed* classes (Fig 8).

## Results

### 1 Batch classification performance

Table 1 contains the individual 10-fold cross-validated accuracy of the classification between the *constant-speed* and the *change-speed* classes. The classification accuracy is above chance level (i.e. 50%) for every subject, with an average of 72.7%.

Table 2 shows the within-subject comparison of the Kappa score associated with the *cued* and *uncued* conditions. Paired sample t-test does not reveal a significant difference (*p* = 0.71).

The Kappa score associated with the subclasses (i.e. 0 → 1, 1 → 2, 2 → 1, 1 → 0) of the *change-speed* class is presented individually in Table 3. Six out of eight subjects are characterized by a significant Kappa (*p* ≤ 0.05) for every subclass, with an agreement level that ranges from fair to almost perfect [54]. The within-subject ANOVA (Table 3) does not reveal a main effect for the ‘subclasses’ factor (*F* = 2.49, *p* = 0.11), which is confirmed by the *Post hoc* test. Nonetheless, we observe that the Kappa score of 0 → 1 is larger than that of 1 → 2 for every subject, except for subject 6, with an average difference of 0.15.

With regards to the *pre-onset* analysis, paired sample t-test suggests that the Kappa scores of the *full-epoch* classifier are significantly larger (*p* ≤ 0.05) than the ones of the *pre-onset* classifier, both for the *cued* (Table 4) and *uncued* (Table 5) conditions.

### 2 Feature visualisation

The k-means clustering of independent components across subjects and cross-validation folds revealed two clusters (Fig 4).

Cluster A accounts for all the subjects, in the sense that at least 40% of the cross-validation folds of each subject have independent components in this cluster. Its scalp map and equivalent dipole location indicate that the independent components belonging to the cluster account for brain activity in the parietal cortex (Table 6). The comparison of the *change-speed* ERSP with the *constant-speed* one, reveals that an event related desynchronization (ERD) occurs when the subjects change speed. The *change-speed* class is characterised by a mu (8–15 Hz) ERD centred at 1 s, and ranging from -1 to 3 s with respect to the speed change onset (0 s). Moreover, we observe a beta (16–31 Hz) ERD starting slightly before the onset and ending at 2 s. However, the largest magnitude of ERD is located in the mu band between 0.5 s and 2 s, which is due to the fact that this phenomenon is consistently the strongest and most repeatable across subjects (S1 Fig).

Cluster B accounts for five subjects, its dipole centroid is located in the primary motor cortex (Table 6), and it displays an ERD pattern similar to that of cluster A.

### 3 Artifact analysis

#### 3.1 EEG correlation

Fig 5 displays, for each subject, the difference between the conditions *standing still*, *constant-speed* and *change-speed*, in terms of correlation (*r* ^2^) among EEG channels. For every combination of conditions, the differences in *r* ^2^ are close to zero at most of the channels. Moreover, the mean absolute difference (m.a.d.) indicates that the average correlation across channels is similar among conditions. Especially, this is true for the difference between *change-speed* and *constant-speed* (last column of Fig 5), where we observe low m.a.d. values. We can conclude that this analysis does not highlight an increased global correlation both for the *constant-speed* and *change-speed* conditions, which suggests that the information used by the classifier is not associated with motion artifacts.

#### 3.2 Comparison of retained and rejected ICs in the time-frequency domain

The individual pairs of retained-rejected ICs with the highest correlation coefficient in the time-frequency domain, are visualised in Fig 6. We observe that for every subject, the pair of ERSPs do not display similar time-frequency patterns.

### 4 Pseudo-online classification performance

In Fig 7 we observe that, for every subject, the ROC curve lies above the chance level line. This means that the true positive rate (TPR) is always larger than the false positive rate (FPR). Based on a four-point categorisation of AUC [55], only subject 3 displays a bad to poor (0.6 ≤ *AUC* ≤ 0.69) classification performance, depending on the amount of consecutive outputs considered (i.e. M1–M3 in Fig 7). Every other subject is characterised by a fair (0.7 ≤ *AUC* ≤ 0.79) to good (0.8 ≤ *AUC* ≤ 0.89) classification performance. Moreover, by increasing the amount of consecutive outputs necessary to detect a gait speed change, we observe a small improvement of AUC, but consistent across all subjects.

The temporal evolution of the decoder output is represented in Fig 8. For every subject, the median of the probability in output is always below threshold 0.5 for the time bins belonging to the *constant-speed* class. On the other hand, there is at least one time bin of the *change-speed* class that is above threshold, for every subject. Moreover, for the subjects with a fair to good AUC, the separation between the two classes can be observed not only for the median but also for the majority of the percentile box. It should be noted that the time bin -1 s of the *change-speed* class, whose window starts at -2 s and ends at 0 s, is characterised by a median close to threshold, in all the subjects.

## Discussion

The 10-fold cross-validated accuracy of the batch EEG classifier, trained to classify between the *change-speed* and the *constant-speed* classes, is above chance level for every subject and on average 72.7% (Table 1). Six out of eight subjects display a classification accuracy larger than 70%, which is usually considered necessary for communication [56]. The performance under the *uncued* condition is not significantly different from the one under the *cued* condition (Table 2), which suggests that the information used by the classifier is not related to the visual cues.

The pseudo-online EEG classification has results that are consistent with the batch analysis, in the sense that subjects with higher batch classification performance display high sliding window AUC (Fig 7). Moreover, we observe that for every subject, the evolution of the decoder output probability (Fig 8) is consistent with the ERSP in Fig 4. Specifically, during the *constant-speed* condition, the decoder output stays under threshold 0.5, while during speed change the output increases above threshold. Given the fair to good sliding window AUC values for the majority of the subjects, we believe that an online implementation of the algorithm is possible.

The batch classification performance associated with the brain activity occurring before the speed change onset (i.e. *pre-onset*) is significantly lower than the one of the *full-epoch* classifier, in both the *cued* (Table 4) and *uncued* (Table 5) conditions. Moreover, in the sliding window analysis, the time bin -1 s preceding the *change-speed* onset is characterised by a median output close to threshold for every subject. We conclude that, with the current dataset and methodology, the brain information subsequent to the speed change onset is crucial for classifying.

Independent components clustering, equivalent dipole localisation and visualisation of the relative scalp maps and time-frequency patterns (Fig 4) reveal that the information used by the classifier is similar among subjects. Cluster A, accounting for brain activity in the posterior parietal cortex of every subject, is characterised by mu and beta ERD, peaking one second after the speed change onset. Such a power perturbation indicates a higher neuronal activity [57] in the posterior parietal cortex (PPC), accompanying the act of accelerating or decelerating. Previous studies have shown that the PPC provides perception of body posture, and helps maintaining normal gait and balance, thus playing an important role in locomotion [58, 59]. Moreover, it is believed that mu and beta ERD in parietal regions are also associated with motor planning [60], and on-line adjustments (i.e. feedforward/feedback conrol) of visually guided upper limb movements [61]. The PPC is also associated with movement intention [62, 63] and motor planning [64] of reaching tasks. It was demonstrated that a single-trial EEG decoder can classify intended movement direction in a delayed saccade-or-reach task, based on features extracted from the PPC [65]. A recent study [7] found that mu and beta band power in premotor and parietal areas is suppressed during conditions that require an adaptation of steps in response to visual input. It is believed that one of the roles of the PPC regards visuomotor transformations required in visually guided motor tasks. The PPC converts the sensory locations of stimuli into the appropriate motor coordinates, or a common reference frame [66], required for making directed movements [67, 68]. Moreover, the PPC not only plays a role in the inverse transformations from sensory information into motor commands, but also in forward ones, such as integrating sensory input with previous and current motor commands to maintain a continuous estimate of the limb state, that can be used to update present and future movement plans [69]. In light of the presented literature, we propose a possible interpretation of the PPC activity found in our study. The mu and beta ERD extends until seconds after the speed change onset, and the *full-epoch* classifier has a significantly higher performance compared with the *pre-onset* one. Consequently, we hypothesise that the PPC is involved in visual input processing during gait adaptation associated with gait speed changes. The observed activation could be also attributed to the neural processes required to align the proprioceptive input to the visual one (i.e. optical flow [70]) in order to constantly update the representation of the body in space [71]. Another explanation could be that the PPC activity during gait acceleration or deceleration may be caused by the discordant visual input (i.e. optical flow), in the transition from stationary to dynamic treadmill walk. For most of the subjects the ERD starts, weakly, before the speed change onset. However, the classification accuracy of the *pre-onset* classifier is significantly above chance level for only a few of them. This pre-onset activity may be due to an occasional step adaptation in preparation to the speed change. Alternatively, motion planning [60] for the *uncued* condition, or cue expectation [72] for the *cued* condition, may have occasionally caused a stronger and detectable activation in the PPC.

Cluster B, accounting for five out of eight subjects, displays a similar, but weaker, ERD perturbation compared to the other cluster, and is located more anteriorly, approximately in the primary motor cortex. This activity might be associated with arm movements, required for balancing in the act of changing speed [57]. Even if subjects were instructed to relax their arms and avoid abnormal movements, it is possible that they occasionally moved their upper limbs for balancing. The involuntary and sporadic nature of this phenomenon would explain the relative weakness of the power perturbation in the ERSP plot. Another possible interpretation for this cluster is that, considering dipole modeling inaccuracies [33, 34], its actual location may be in the premotor cortex. This would be in agreement with recent findings [7, 10], suggesting that the premotor cortex may have a role in the motor planning involved in gait initiation and adaptation.

The majority of the subjects displays significant Kappa scores for every type of speed change (Table 3). Moreover, the within-subject ANOVA and *Post hoc* test suggest that the subclasses are characterized by comparable classification performances. Nonetheless, in every subject excluding subject 6, the Kappa score of walk initiation (i.e. 0 → 1) is larger than the one of acceleration from 1 to 2 Km/h (i.e. 1 → 2). We investigate this observation by visualizing the features of the two subclasses in S2 Fig. The magnitude of the ERD and the baseline power associated with 1 → 2 are smaller, compared respectively with the ones of 0 → 1. This may be caused by the different baseline levels of the two subclasses: it has been shown [6] that the rest condition (i.e. 0 Km/h) is characterized by a spectral power in the mu and beta bands that is significantly larger than the one of the active walk condition. However, the classification performance of 1 → 2 is above chance level for six subjects, meaning that the time-frequency pattern is still informative for the purpose of classifying. These considerations lead to the conclusion that the four types of speed change share a common feature space, and confirm that the activity observed in the PPC is associated with the online adaptation of gait, instead of the abstract concept of speed, slowing down or speeding up. In order to validate this interpretation, future studies should focus on detecting gait acceleration or deceleration with smaller differences between the initial and final speed, and possibly define a lower bound for detectability.

Since PCA is performed before classification, the time-frequency patterns visualized in Fig 4 are not the actual feature vectors used by the classifier. This visualization approach is motivated by the fact that clustering across subjects and visualization of principal components (PCs) would have been counterintuitive. However, in order to clarify the role of PCA, in S3 Fig we visualize the principal components for a batch cross-validation fold of only one subject. From this analysis we observe that the time-frequency features accounted by the most important PCs are consistent with the mu and beta ERD visualized in Fig 4.

In the artifact analysis, the fact that the *constant-speed* and *change-speed* conditions are not characterized by a strong correlation across channels suggests that the information used by the classifier is not associated with motion artifacts. Moreover, we showed that the pairs of rejected-retained ICs, with the largest correlation coefficient in the time-frequency domain, do not display any similarity for every subject (Fig 6). We hypothesise that if the retained ICs were contaminated by external noise, we would have observed similar time-frequency patterns, with higher magnitude in the rejected ICs. These considerations lead to the conclusion that the neural information was well separated from artifacts, meaning that the features used by the classifier can be considered reliably clean. A possible limitation of this validation technique is that, with regards to shocks undergone by the head, it may be less rigorous than one based on coherence analysis between the EEG and vertical acceleration (e.g. similar to [17]). However, vertical shocks are not the only artifact affecting the EEG signal during walking; therefore, we believe that our approach is more general, in the sense that it checks for the presence of any type of artifact, from neck and face muscle activation to general motion artifacts. Nonetheless, in future studies it will be important to add an accelerometer on the subjects’ head in order to systematically, and more completely, check for head shock artifacts. Moreover, the information from the accelerometer could be regressed out from the EEG, even though it may be challenging given the different frequencies of the two signals.

A recent study [73] demonstrated the feasibility of detecting the intention of gait initiation from movement related cortical potentials (MRCP), on a single trial basis. During the preliminary phase of our study, we tried to include the EEG delta band in the analysis, however the classifier did not gain any benefit. A possible explanation is that, in the current experimental setup, gait speed changes are performed even after movement (i.e. gait) initiation (e.g. 1 → 2), making the MRCP not detectable. These considerations, added to the high probability of motion artifacts at low frequencies [17, 18], motivated a more focused analysis in the frequency band 8–40 Hz.

Yuan et al. [74] decoded imagined and executed hand clenching speed, based on EEG alpha and beta activities in the sensorimotor cortex. Currently, we are not able to find a similar activity for locomotion speed. One possible explaination is that walking requires a complex activation and coordination of both leg and arm muscles [75], which may be more challenging to decode than the frequency of single-hand clenching. Moreover, in our experiment we did not pace the locomotion speed by a metronome, and subjects could change speed by increasing either the stride length or the stepping frequency. Finally, hand clenching at high frequency is a less automated movement compared to locomotion [76], therefore we believe that it would require a larger involvement of the sensorimotor cortex. Given these considerations, we suppose that, before reproducing the findings of Yuan et al. [74] for locomotion, future studies should decode the frequency of a single leg movement; and, if successful, extend to more complex paradigms where locomotion is paced by a metronome and either stride length or stepping frequency are fixed.

Given the timing of the observed phenomenon, BMI applications of the proposed classifier are currently limited at monitoring, rather than predicting, speed changes. Future studies will investigate a possible application of the proposed classifier and experimental paradigm in top-down robotic neuro-rehabilitation after stroke [77]. In particular, the brain activity may be monitored, during robotic-assisted gait acceleration/deceleration, to assess functional improvements [6], and give neuro-feedback to the patient in order to increase voluntary drive [78–82], active participation and, therefore, improve motor learning [83, 84]. It will be necessary to investigate whether robotic-guided (i.e. passive) speed changes would still activate the posterior parietal cortex. We hypothesise that, depending on the subject’s participation to the rehabilitation task, the PPC would still be performing the visual transformations from the sensory locations of visual stimuli (e.g. optical flow) into a common reference frame [66]. However, patients with gait deficits or neurological lesions may achieve a lower detection performance compared to healthy subjects. If future studies will highlight such a performance drop, it will be worth focusing on a co-adaptive BCI paradigm, that has proven effective also with users with severe motor impairment [85]. Furthermore, in rehabilitation paradigms that do not include a robotic device, it is unlikely that patients would be able to accelerate or decelerate between 1 and 2 Km/h, therefore, as previously stated, it may be of interest to investigate the detectability of milder speed changes. Moreover, future studies should focus on the pre-onset activity, and investigate the possibility of teaching to the subjects to modulate it, as currently done for motor imagery by neuro-feedback [86]. This could allow us to predict the speed change onset, before it actually happens. If such studies will second this idea, new pathways towards causal and natural BCI real-time control of an exoskeleton robot may be opened.

The contributions of this work are summarized in the following paragraph. To the authors’ knowledge, this is the first study that analyses the brain activity during volitional gait acceleration and deceleration at various speeds in treadmill walking. The main finding was that, as subjects change their gait speed, mu and beta rhythms are suppressed (i.e. increased activity) in parietal areas. This result was obtained by analyzing the features used by a classifier that detects gait speed changes from the EEG signal. We observed that the strongest and most repeatable brain activity occurs 1 s after the speed change onset, which suggests that the observed PPC activation may reflect motor planning, visuomotor transformations, or proprioceptive feedback processing during online gait adaptation. In conclusion, we proposed future research directions, in order to make the classifier applicable in BMI-based rehabilitation robotics.

## Conclusion

To the authors’ knowledge, this is the first study showing that mu and beta rhythms in the PPC are suppressed during gait speed changes on a treadmill, and that a single-trial EEG classifier is able to detect them. The observed cortical activation may reflect motor planning and visuomotor transformations during online gait adaptation. As stated throughout the Discussion, this work leaves some open questions regarding the experimental conditions in which the gait speed detection is feasible. However, we hope that the findings of this study may contribute to a better understanding of the neural mechanisms underlying human gait control, and encourage future research aiming to develop a more natural and intuitive brain-computer interface for applications in post-stroke gait rehabilitation.

## Supporting Information

S1 Fig ERD quantification. For each member of the clusters in Fig 4, we quantify mu (i.e. 8–15 Hz) and beta (i.e. 16–31 Hz) ERD in the pre-onset and post-onset time frames. The time regions of interest (ROI) are selected by visual inspection of Fig 4. Specifically, the post-onset ROI is centred around 1 s and is 1 s long. The pre-onset ROI is the last 1 s of the pre-onset time-frequency features (i.e. -1.211 s and -0.211 s). The beta band is shortened between 16 and 25 Hz, based on visual inspection of Fig 4. The ERD [57] for a given frequency band is computed by the formula *ERD*(%) = (*A*−*R*)/*R*×100, where *A* is the mean power in a given time frame (e.g. pre-onset or post-onset), while *R* is the mean power in the reference period, which we set as the first 1 s of the epoch. We observe that the post-onset mu ERD has the strongest and most consistent magnitude across subjects, and that the pre-onset ERD is consistently smaller than the post-onset one. It should be noted that Cluster B does not contain subjects 5,6 and 8.
(TIF)

S2 Fig Feature visualization of subclasses. The clusters presented in Fig 4 are analysed with respect to the subclasses 0 → 1 and 1 → 2, excluding subject 6. The methodologies are equivalent to the ones used for Fig 4, with the only exception that the ERSPs of the two subclasses are baselined using the first 2.8 s (i.e. from -3789 to -1007 ms) of the 0 → 1 subclass to highlight possible differences. We observe that the ERSP of the 0 → 1 class displays larger ERD magnitude and baseline power with respect to the 1 → 2 class.
(TIF)

S3 Fig Principal components visualization. Principal components and the respective eigenvectors are visualized, along with the associated ERSPs and IC, for one cross-validation fold of Subject 7. The automatically selected IC, as explained in sections 4.4 and 4.6, is represented in column A. The ERSPs associated with the two classes are presented in column B. In order to visualize only the most important Principal Components, the Scree Test Acceleration Factor [37] of the eigenvalues is computed. The eigenvectors associated with the selected eigenvalues are shown, reshaped to fit the time-frequency representation, in column C. This representation describes how areas of the time-frequency domain are weighted in order to obtain the principal components (PCs). The projection of each single-trial spectrogram onto the eigenvectors results in the single-trial principal components, plotted in column D. PCs are visualized separately for the training and test set, and colored according to their class. The IC and ERSP reflect the increased activity in the posterior cortex associated with the speed change, which is in accordance with the clusters in Section 2. The first eigenvector, in column C, accounts for the variability in the whole *mu* band, which may be associated with the power decrease during gait as opposed to a rest condition that we observed in S2 Fig and that was found in previous studies [6]. Moreover, we observe that the weights of the first eigenvector are stronger in the *mu* band after 0 s, which may account for the ERD elicited by the change of speed. Similarly, the second eigenvector weights the mu and beta bands negatively prior to 0 s and positively after 0 s. Given these observations, the projections of trials characterized by an ERD (i.e. *change-speed*) should have a smaller magnitude in both PC1 and PC2 axis as compared to trials without ERD (i.e. *constant-speed*). This is confirmed by the plots in column D, where the *change-speed* trials lie in the left-lower quadrant of the PC1-PC2 plot, while the *constant-speed* ones are in the right-upper quadrant.
(TIF)
