# Supplementary figures and images for: EEG Single-Trial Detection of Gait Speed Changes during Treadmill Walk
Source: PLoS One. 2015 May 1;10(5):e0125479. doi: 10.1371/journal.pone.0125479 (PMC4416798; doi:10.1371/journal.pone.0125479)

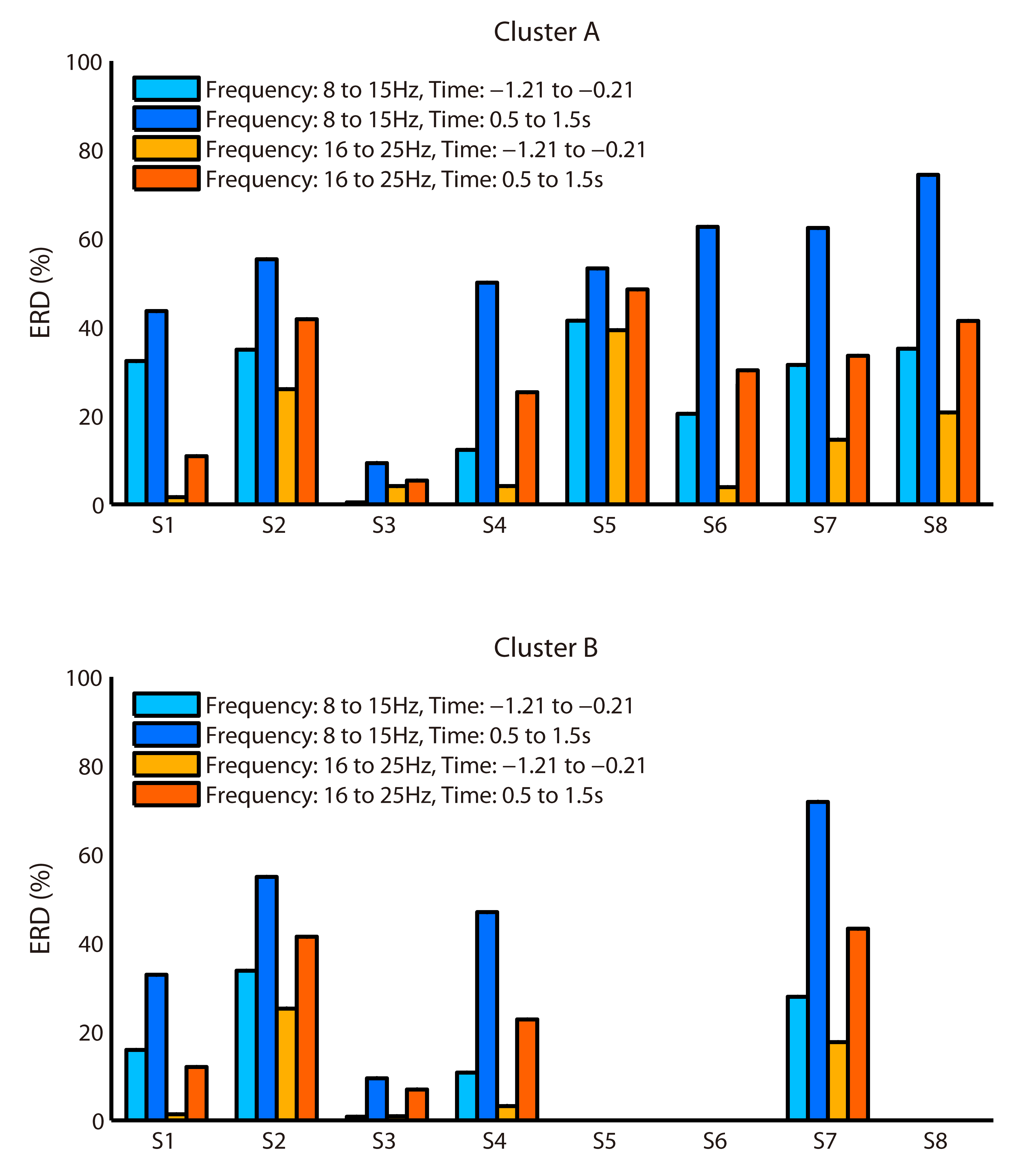

Supplement: S1 Fig — For each member of the clusters in Fig 4, we quantify mu (i.e. 8–15 Hz) and beta (i.e. 16–31 Hz) ERD in the pre-onset and post-onset time frames. The time regions of interest (ROI) are selected by visual inspection of Fig 4. Specifically, the post-onset ROI is centred around 1 s and is 1 s long. The pre-onset ROI is the last 1 s of the pre-onset time-frequency features (i.e. -1.211 s and -0.211 s). The beta band is shortened between 16 and 25 Hz, based on visual inspection of Fig 4. The ERD [57] for a given frequency band is computed by the formula ERD(%) = (A−R)/R×100, where A is the mean power in a given time frame (e.g. pre-onset or post-onset), while R is the mean power in the reference period, which we set as the first 1 s of the epoch. We observe that the post-onset mu ERD has the strongest and most consistent magnitude across subjects, and that the pre-onset ERD is consistently smaller than the post-onset one. It should be noted that Cluster B does not contain subjects 5,6 and 8. (TIF) [file pone.0125479.s001.tif]

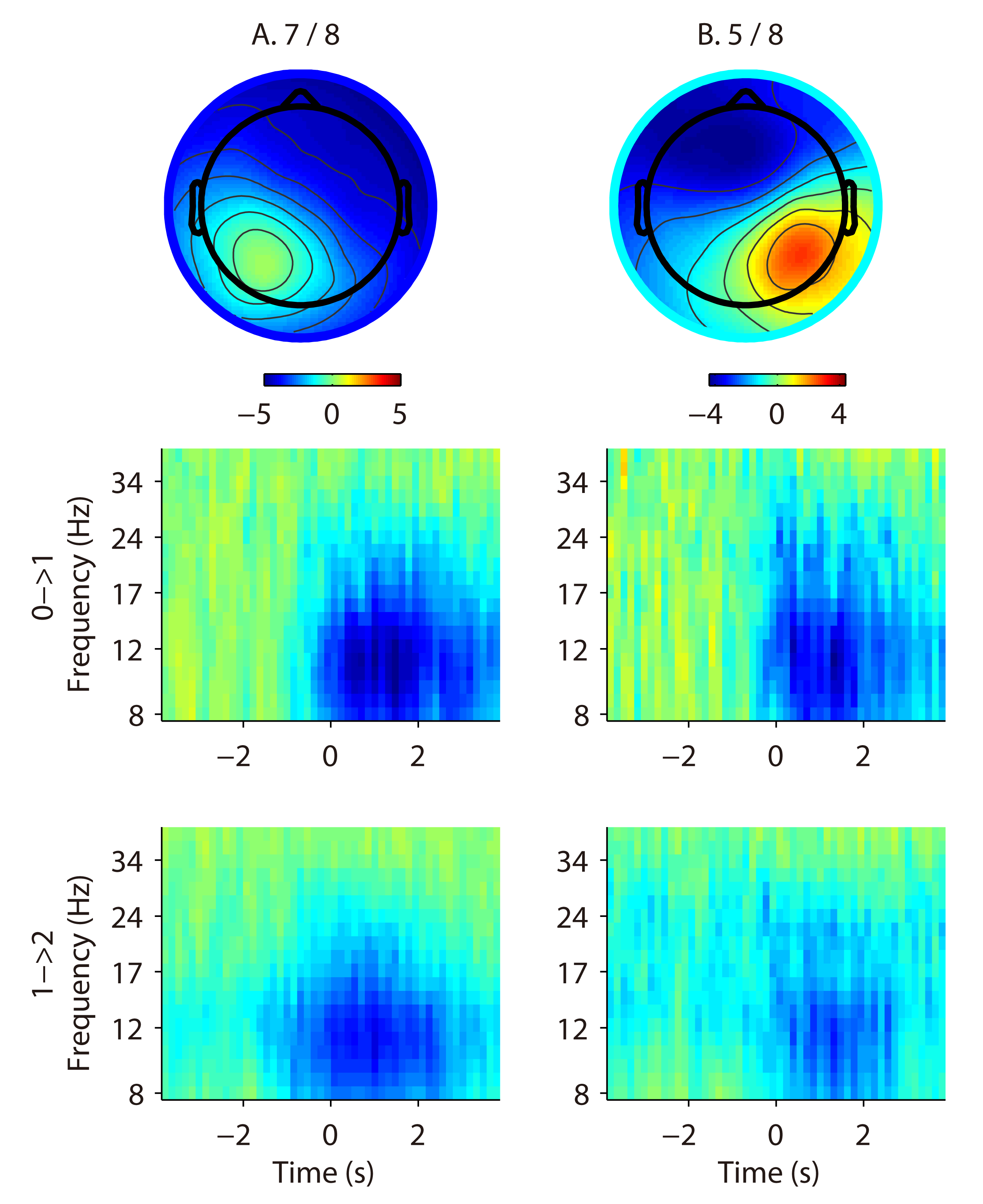

Supplement: S2 Fig — The clusters presented in Fig 4 are analysed with respect to the subclasses 0 → 1 and 1 → 2, excluding subject 6. The methodologies are equivalent to the ones used for Fig 4, with the only exception that the ERSPs of the two subclasses are baselined using the first 2.8 s (i.e. from -3789 to -1007 ms) of the 0 → 1 subclass to highlight possible differences. We observe that the ERSP of the 0 → 1 class displays larger ERD magnitude and baseline power with respect to the 1 → 2 class. (TIF) [file pone.0125479.s002.tif]

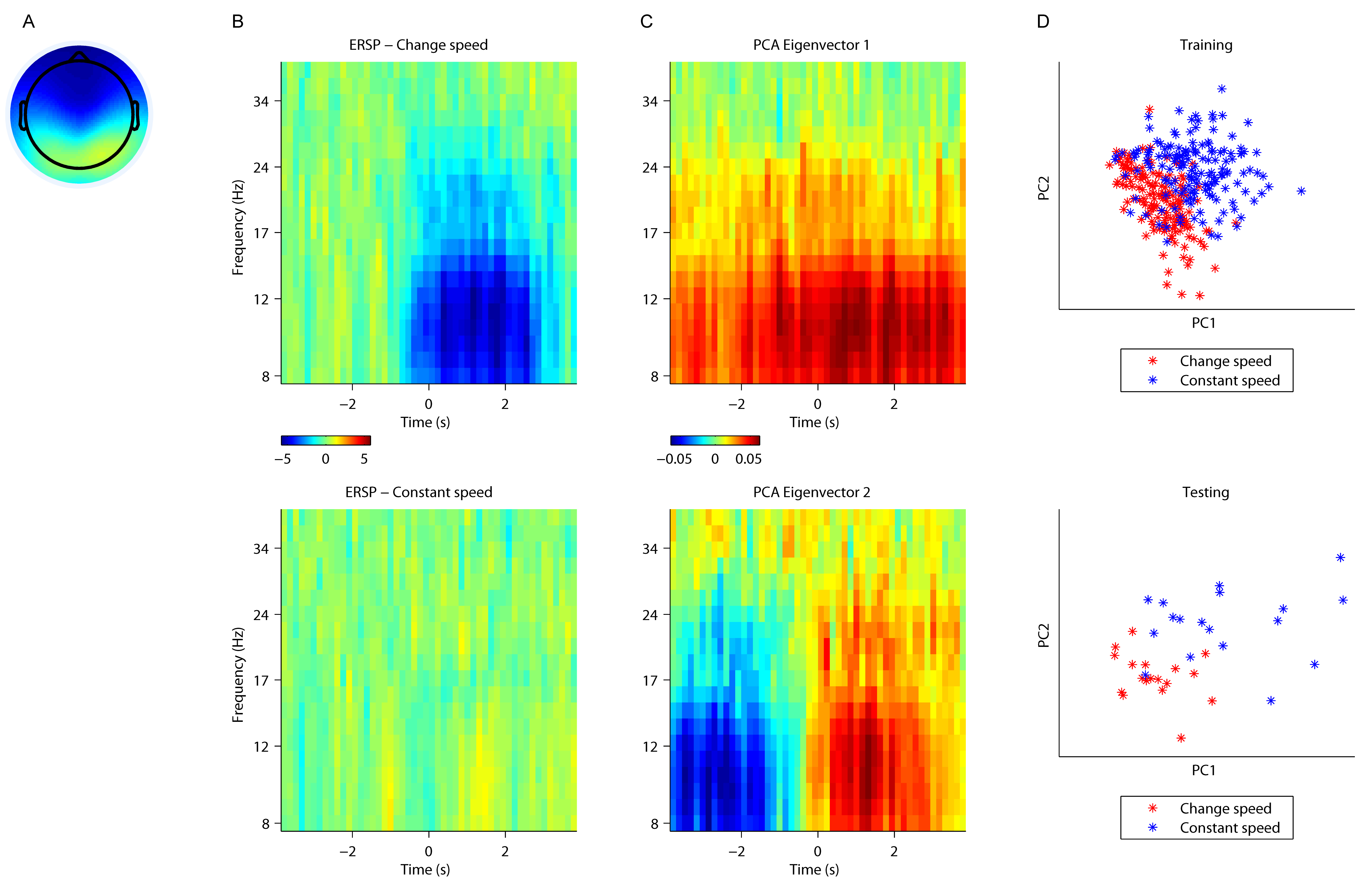

Supplement: S3 Fig — Principal components and the respective eigenvectors are visualized, along with the associated ERSPs and IC, for one cross-validation fold of Subject 7. The automatically selected IC, as explained in sections 4.4 and 4.6, is represented in column A. The ERSPs associated with the two classes are presented in column B. In order to visualize only the most important Principal Components, the Scree Test Acceleration Factor [37] of the eigenvalues is computed. The eigenvectors associated with the selected eigenvalues are shown, reshaped to fit the time-frequency representation, in column C. This representation describes how areas of the time-frequency domain are weighted in order to obtain the principal components (PCs). The projection of each single-trial spectrogram onto the eigenvectors results in the single-trial principal components, plotted in column D. PCs are visualized separately for the training and test set, and colored according to their class. The IC and ERSP reflect the increased activity in the posterior cortex associated with the speed change, which is in accordance with the clusters in Section 2. The first eigenvector, in column C, accounts for the variability in the whole mu band, which may be associated with the power decrease during gait as opposed to a rest condition that we observed in S2 Fig and that was found in previous studies [6]. Moreover, we observe that the weights of the first eigenvector are stronger in the mu band after 0 s, which may account for the ERD elicited by the change of speed. Similarly, the second eigenvector weights the mu and beta bands negatively prior to 0 s and positively after 0 s. Given these observations, the projections of trials characterized by an ERD (i.e. change-speed) should have a smaller magnitude in both PC1 and PC2 axis as compared to trials without ERD (i.e. constant-speed). This is confirmed by the plots in column D, where the change-speed trials lie in the left-lower quadrant of the PC1-PC2 plot, while the c [file pone.0125479.s003.tif]
